# *Salvia officinalis* L. and *Salvia sclarea* Essential Oils: Chemical Composition, Biological Activities and Preservative Effects against *Listeria monocytogenes* Inoculated into Minced Beef Meat

**DOI:** 10.3390/plants12193385

**Published:** 2023-09-25

**Authors:** Boutheina Ben Akacha, Anis Ben Hsouna, Ivana Generalić Mekinić, Améni Ben Belgacem, Rania Ben Saad, Wissem Mnif, Miroslava Kačániová, Stefania Garzoli

**Affiliations:** 1 Laboratory of Biotechnology and Plant Improvement, Centre of Biotechnology of Sfax, B.P “1177”, Sfax 3018, Tunisia; akachabouthaina@gmail.com (B.B.A.); benhsounanis@gmail.com (A.B.H.); amanibelgassem@gmail.com (A.B.B.); raniabensaad@gmail.com (R.B.S.); 2 Department of Environmental Sciences and Nutrition, Higher Institute of Applied Sciences and Technology of Mahdia, University of Monastir, Monastir 5000, Tunisia; 3 Department of Food Technology and Biotechnology, Faculty of Chemistry and Technology, University of Split, R. Boškovića 35, HR-21000 Split, Croatia; gene@ktf-split.hr; 4 Department of Chemistry, College of Sciences at Bisha, University of Bisha, P.O. Box 199, Bisha 61922, Saudi Arabia; wmoneef@ub.edu.sa; 5 Faculty of Horticulture, Institute of Horticulture, Slovak University of Agriculture, Tr. A. Hlinku 2, 949 76 Nitra, Slovakia; miroslavakacaniova@gmail.com; 6 Department of Chemistry and Technologies of Drug, Sapienza University, P. le Aldo Moro, 5, 00185 Rome, Italy

**Keywords:** *Salvia*, chemical composition, natural preservatives, food pathogens, minced meat, refrigerated storage

## Abstract

In this study, *Salvia officinalis* L. and *Salvia sclarea* essential oils (EOs) were investigated using gas chromatography-mass spectrometry (GC-MS) to describe their chemical composition. The obtained results show, for both EOs, a profile rich in terpene metabolites, with monoterpenes predominating sesquiterpenes but with significant qualitative and quantitative differences. The main compound found in the *Salvia officinalis* EO (*SO*EO) was camphor (19.0%), while in *Salvia sclarea* EO (*SC*EO), it was linalyl acetate (59.3%). Subsequently, the in vitro antimicrobial activity of the EOs against eight pathogenic strains was evaluated. The disc diffusion method showed a significant lysis zone against Gram-positive bacteria. The minimum inhibitory concentrations (MICs) ranged from 3.7 mg/mL to 11.2 mg/mL, indicating that each EO has specific antimicrobial activity. Both EOs also showed significant antiradical activity against DPPH radicals and total antioxidant activity. In addition, the preservative effect of *SO*EO (9.2%) and *SC*EO (9.2%), alone or in combination, was tested in ground beef, and the inhibitory effect against *Listeria monocytogenes* inoculated into the raw ground beef during cold storage was evaluated. Although the effect of each individual EO improved the biochemical, microbiological, and sensory parameters of the samples, their combination was more effective and showed complete inhibition of *L. monocytogenes* after 7 days of storage at 4 °C. The results show that both EOs could be used as safe and natural preservatives in various food and/or pharmaceutical products.

## 1. Introduction

The name *Salvia* is derived from the Latin word “*salvare*” meaning “to heal” or “to be healthy”, which summarizes the popular belief in its “magical” health-promoting properties and its frequent use in folk medicine to treat various types of ailments [1,2].

The plants of the genus *Salvia*, which is the largest in the Lamiaceae family with over 900 species, are distributed worldwide, including the Mediterranean region, Central Asia, the Pacific Islands, tropical Africa, and the Americas. Numerous *Salvia* species are of commercial importance as spices and flavorings in perfumery and cosmetics [1,3]. They are known for their attractive colored flowers, which are usually pink to red or purple to blue [4,5].

*Salvia sclarea* is a biennial herbaceous plant or short-lived perennial with an upright habit. It can grow up to 1.60 m high when in flower. It is quite hardy and tolerates temperatures down to approximately −15 °C. The deciduous foliage is silver-gray and has long, fragrant, and oval leaves [5,6] that can grow up to 15 cm long. The purple-blue, 3 cm long flowers, grow on the main stem and on the secondary branches, forming a panicle about 60 cm long. The essential oil (EO) of *Salvia sclarea* (*SC*EO) is often used as a flavoring in the food industry, which is why the plant is widely cultivated commercially [7].

The therapeutic and beneficial health properties of plants have attracted great interest in the course of scientific developments because of their low toxicity, pharmacological effects, and economic profitability [8,9,10,11]. Most studies have focused on the benefits of plant-derived phytochemical compounds and their positive effects on human health. Naturally derived additives from plants may be single compounds or groups of compounds (mixtures), as in the case of EOs [12,13,14,15,16]. Recently, the food industry has shown great interest in natural compounds added directly or in combination with other compounds to achieve a synergistic effect [17,18]. It has been reported that the direct addition of EOs and aromatic plant extracts to foods induces antioxidant or antimicrobial effects [19]. In particular, special attention has been paid to EOs because of their antiradical properties [20,21].

*Salvia* EOs have significant activities, such as antimicrobial, antioxidant, anticholinesterase, cognitive and mood enhancing, work-related stress reduction, antimutagenic, anticancer, anti-inflammatory, and choleretic [21,22,23,24,25,26,27,28,29]. However, the EO chemical composition of medicinal and aromatic plants is strongly influenced by various genetic and environmental factors [30,31].

The aim of this study was to chemically investigate EOs extracted from the leaves of *SO*EO and *SC*EO grown in central Italy and to evaluate their biological effects in terms of antioxidant and antimicrobial activity against a range of foodborne pathogenic and spoilage bacteria. In addition, the inhibitory effect against *L. monocytogenes* was studied for the first time to test their preservative effect in ground beef. For this purpose, the changes in ground beef meat after treatment with *SC*EO and *SO*EO, alone and in combination, and the effects on microbiological, oxidative/lipid stability and sensory properties were evaluated. On this basis, chemometric analysis and principal component analysis (PCA) were used as multivariate analytical techniques to study the effects of the treatments on the shelf-life of the meat. Finally, a heat map was created to distinguish the investigated samples according to different storage periods, and to understand the relationships among the evaluated parameters.

## 2. Results and Discussion

### 2.1. GC-MS Analyses

The results of the analyses performed using the GC-MS technique showed the presence of forty-seven components: thirty-five in *SO*EO and twenty-eight in *SC*EO (Table 1). In general, for both EOs, the monoterpene content exceeded that of sesquiterpene, although the chemical profiles were quite different. In *SO*EO, the main compounds were camphor (19.0%), *β*-pinene (14.5%), *α*-thujone (12.9%), and humulene (11.9%), and in *SC*EO, linalyl acetate was the most abundant component with 59.3%, followed by linalool (11.3%) and germacrene D (10.5%). In addition, a number of molecules with percentage mean values ranging from 0.1 to 6.9% characterized the volatile profiles of the two EOs differently.

The GC chromatograms of *SO*EO and *SC*EO are reported in Figures S1 and S2, respectively.

Our results are in agreement with those reported by other authors. Thus, linalyl acetate was the major compound (over 50%) in clary sage EO from Slovakia, Bulgaria, Serbia, and Italy [32,33,34,35].

As for *SO*EO, the presence of camphor as a predominant constituent was consistent with what has been reported in previous studies on EOs from the leaves of plants grown in Tunisia and Slovakia [36,37]. In contrast, Porte et al. [38] reported a higher content of *α*-thujone (40.90%) than camphor (26.12%). Remarkably, in our sample, the presence of a considerable amount of *β*-pinene (14.5%) on average higher than in previous works was detected. Also, *β*-pinene was found in a considerable amount (14.5%), higher than previously reported. However, a variability in the *β*-pinene content was found depending on the growth area of the plant [39].

It has long been known that terpenes and terpenoids play a role in the treatment of various types of diseases, thanks to their diverse activities, such as anticancer, antimicrobial, anti-inflammatory, antioxidant, and analgesic [40].

Regarding the biological activity of the main monoterpene compounds in the two EOs, previous studies have reported that camphor has weak antimicrobial activity [41,42]. For example, Greek sage (*Salvia fruticosa*) EO, which contains camphor as a major constituent, showed low activity against various bacteria, such as *Escherichia coli*, *Pseudomonas aeruginosa*, *Salmonella typhimurium*, *Staphylococcus aureus*, *Rhizobium leguminosarum*, and *Bacillus subtilis.* However, Viljoen et al. [43] reported a synergistic antimicrobial effect between camphor and 1,8-cineole.

On the other hand, camphor is known to be toxic when ingested [44], and it has also been reported that the toxicity of *SO*EO is related to its presence in large quantities [45].

*α*- and *β*-Pinene have been found in the EOs of many plants, and a wide range of its pharmacological activities have been reported. In particular, the antimicrobial activities of pinene isomers and enantiomers against several bacterial and fungal cells were evaluated showing that only the positive enantiomers were active [46].

Linalool, which is widely used in the perfume industry, is a monoterpene alcohol that also exhibits various biological properties, such as antioxidant, antimicrobial, anti-inflammatory, and anticancer [47]. Linalyl acetate, the acetate ester of linalool, is also reported to have anti-inflammatory [48] and antimicrobial activities [49].

In our work, the major sesquiterpenes were germacrene D (10.5%) and humulene (11.9%) in *SC*EO and in *SO*EO, respectively. These two compounds have been reported to have good antibacterial activity [50,51].

### 2.2. Antioxidant Capacities of SOEO and SCEO

The antioxidant capacity of the tested EOs was evaluated by DPPH free radicals scavenging and phosphomolybdenum assays, and the results were expressed as 50% inhibition concentration (IC_50_) for both assays. BHT was used as the standard for the DPPH method and gallic acid for the phosphomolybdenum assay.

Both EOs showed significant antioxidant activity compared to BHT. The IC_50_ results for the DPPH assay showed an antioxidant activity of BHT < *SO*EO < *SC*EO with IC_50_ values of 43.85 ± 0.87, 38.89 ± 0.93, and 27.67 ± 0.98 µg/mL, respectively. The results obtained are in agreement with those reported by Tosun et al. [52] for eight *Salvia* species from Turkey, with IC_50_ values ranging from 15.2 to 88 µg/mL.

The antioxidant capacities of EOs through the formation of the green phosphomolybdenum complex were also measured using the absorbance intensity. As in the DPPH assay, *SC*EO was the most effective with an IC_50_ value of 123 ± 0.99 µg/mL, followed by gallic acid (196.0 ± 0.60 µg/mL) and *SO*EO (595.5 ± 0.97 µg/mL). Our results are in agreement with previous reports [53,54,55].

### 2.3. Antibacterial Properties of SCEO and SOEO

The disc diffusion test was used to measure the antimicrobial activity of the samples, which were classified into two groups according to the diameter of the zone of inhibition: (1) zero, i.e., no activity, and (2) presence of activity between 8 and 25 mm in diameter. Among the investigated EOs, *SC*EO showed greater antibacterial activity against the Gram-positive strains compared with *SO*EO, considering the larger zones of inhibition. Specifically, five Gram-positive bacteria were tested (Table 2), including *B. cereus* and *L. monocytogenes*, which did not respond to the effect of *SO*EO at the two concentrations tested. *SO*EO also showed the lowest antibacterial activity against *E. faecalis*.

Gram-positive bacteria were more sensitive to both EOs than Gram-negative bacteria, which were resistant to the concentrations tested (25.0 µg/mL and 50.0 µg/mL) with the exception of *SO*EO-sensitive *S. enterica*.

The mechanisms of the antibacterial action of EOs are thought to be increased cell permeability due to the hydrophobicity of EO [56,57] and their toxic effects on membrane structure and function [58]. It has also been previously observed that EOs exhibit higher activity against Gram-positive than Gram-negative bacteria, which is due to the presence of an outer membrane that restricts the diffusion of EO components [18]. On the other hand, the peptidoglycan cell wall in Gram-positive bacteria offers less resistance to hydrophobic compounds [15,59].

The antimicrobial activity of the samples was investigated using a broth microdilution susceptibility test against eight bacterial strains known as major food contaminants. The results shown in Table 3 indicate that *SO*EO was effective against all eight strains tested, with MIC values ranging from 4.6 to 7.5 mg/mL. This is comparable to the work of Longaray Delamare et al. [22]. Very weak effects against *E. coli* and *P. aeruginosa* can be attributed to the bacterial strains and the different chemical constituents of the EOs tested.

The MBC/MIC values were calculated to determine the effect of the EOs against bacteria. *SO*EO showed bactericidal activity against almost all strains used in this study, except *Enterococcus faecalis* and *L. monocytogenes*. These data are in agreement with the results of Adrar et al. [60], who tested the antibacterial activity of *Salvia officinalis* EO alone and in combination with *Thymus numidicus*.

*SC*EO showed similar results to *SO*EO, except for *E. coli*, which was more sensitive to the effect of *SC*EO, with an MIC value of 3.7 mg/mL. In addition, *SC*EO showed bacteriostatic activity against *L. monocytogenes* and *S. enterica* and bactericidal activity against the other strains tested. All this indicates that *SC*EO is an effective bacterial inhibitor and bactericide with a broad antibacterial spectrum.

Cui et al. [61] demonstrated that *Salvia sclarea* EO had several antibacterial effects in the order *S. aureus* = *Klebsiella pneumonia* > *P. aeruginosa*. There are several reasons for the discrepancies between our results and the previous report: (a) the difference between species; (b) different extraction methods of EOs; (c) probable differences between strains of the same origin, which are the result of long adaptation to the ecological environment, artificial selection and crossbreeding [62].

A previous study reported the antimicrobial and antifungal effect of *Salvia sclarea* EOs against ten bacterial and four fungal species, respectively [63].

According to the literature, there is a relationship between the composition of EOs and their antibacterial activity [64]. In general, part of the antimicrobial activity is attributed to oxygenated terpenoids (e.g., phenolic alcohols and terpenes). For example, *α*-pinene (a monoterpene hydrocarbon) and borneol (an oxygenated monoterpene) and other minor constituents of *Salvia officinalis* and *Salvia triloba* EOs, have been attributed antimicrobial activity [22].

Although food testing is still required, the results of antibacterial activity suggest that both EOs can be considered natural alternatives to “traditional food preservatives”, as they were effective against investigated foodborne pathogens, thus contributing to food safety.

### 2.4. Application of SOEO, SCEO Alone and in Combination for Preservation of Minced Beef Meat during 14 Days of Refrigerated Storage

EOs have pronounced antimicrobial and preservative effects, as they consist of a different active compound (e.g., terpenes, terpenoids, carotenoids, coumarins, and curcumins) that are of great importance in the food sector. For this purpose, 9.2% (*SC*EO), 9.2% (*SO*EO), and 9.2 + 9.2% for the mixture of *SC*EO and *SO*EO were added to the raw ground beef, which corresponds to 2 times the MIC against *L. monocytogenes* ATCC 19117.

#### 2.4.1. Chemical Stability Changes

pH has a strong influence on water holding capacity (WHC), which is closely related to product yield and meat quality [65]. Changes in meat pH are the result of postmortem metabolism (glycolysis) and the conversion of glycogen to lactic acid. Variations in the rate and/or extent of postmortem glycolysis are responsible for much of the variation in WHC and meat color, so it is important to monitor pH change as a function of time [66].

The pH results of five batches stored at 4 °C for 14 days are shown in Table 4. The initial pH of the minced beef meat was similar in all five batches; an exponential increase was observed in the control sample, reaching 7.85 ± 0.05 after 14 days, while the BHT reached only 6.96 ± 0.08 at the end of storage.

Our treatments with *SC*EO and *SO*EO at 9.2% showed the best results, both alone and in combination, with a positive synergistic effect observed when the two oils were mixed. The pH did not exceed the upper limit established by Ripke Ferreira et al. [67], who stated that pH is an indicator of meat quality and should be between 5.6 and 6.2. An increase in pH promotes the rapid growth of pathogenic microorganisms, especially psychrophilic microorganisms. Therefore, ground beef treated with *SO*EO + *SC*EO can be considered fit for human consumption. Our results are in agreement with those of Ripke Ferreira et al. [67], who demonstrated that the addition of *S. officinalis* EO lowered the pH value in salmon burgers more slowly than the control product. This conservative effect is closely related to the demonstrated antioxidant and antibacterial abilities of *S. officinalis* EO [22,60].

**Figure 1:**
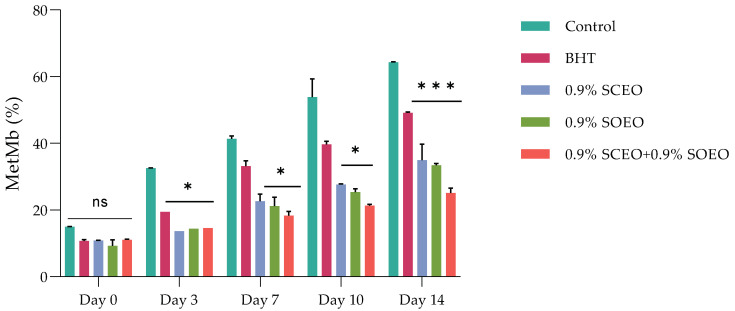
Effect of treatment and refrigerated storage time on metmyoglobin (MetMb) formation (%); ± standard deviation (SD) of the three replicates. Groups (BHT, 0.9% *SC*EO, 0.9% *SO*EO, 0.9% *SC*EO + 0.9% *SO*EO) vs. group (control): *** *p* ≤ 0.001, ** *p* ≤ 0.01, * *p* ≤ 0.05.

The most important factors contributing to meat color are the myoglobin content, the chemical state of the heme structure, and the pH of the meat [68]. It has been shown that the myoglobin content depends mainly on the species and age of the animal, and that the pH of the muscle is mainly related to the biochemical state of the muscle at the time of slaughter and after the development of rigor mortis [11,69,70]. These two factors contribute to the color of the meat and the occurrence of color defects. Metmyoglobin (MetMb) production was followed over the five-day follow-up period for each sample, and the results are shown in Figure 1.

Color is the consumer’s first impression of the quality of a meat product, and is therefore of fundamental importance. Fortunately, meat color can be adjusted if you know the various factors that influence it. The color of fresh and cured meat depends largely on the myoglobin content [70,71,72]. It consists of a protein component and a non-protein porphyrin band with an iron atom in between, which plays a key role in determining meat color [73,74].

**Figure 2:**
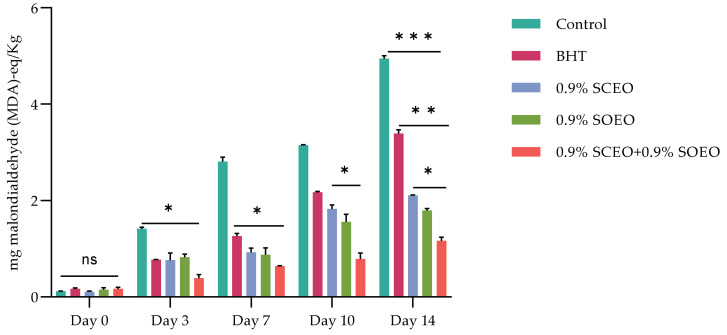
Effect of BHT, *Salvia officinalis* (*SO*EO), and salvia sclarea (*SC*EO) essential oils and their combination (*SC*EO + *SO*EO), on TBARS evolution (mg malondialdehyde (MDA)-eq/Kg) of minced beef stored at 4 °C for 14 days; ± standard deviation (SD) of the three replicates. Groups (BHT, 0.9% *SC*EO, 0.9% *SO*EO, 0.9% *SC*EO + 0.9% *SO*EO) vs. group (control): *** *p* ≤ 0.001, ** *p* ≤ 0.01, * *p* ≤ 0.05.

The effects of *SC*EO, *SO*EO and their mixture on MetMb formation in minced meat during 14 days of storage are shown in Figure 2.

The two EOs alone significantly (*p* < 0.05) inhibited the accumulation of MetMb and thus the effects associated with the third day of storage. However, the mixture of the two EOs was more effective in inhibiting MetMb formation than the oils alone.

Ruedt et al. [75] demonstrated that the main cause of red meat discoloration is oxidation of oxymyoglobin (light red) to MetMb (brown). It has been reported that oxidation products of lipids accelerate the oxidation of myoglobin [76]. In the present study, the results show that treatment with the combination of EOs compared to the two EOs alone was more effective in controlling both lipid oxidation and MetMb formation (Figure 2). The TBARS values of the treated and untreated samples increased progressively during cold storage, reaching a concentration of 3.15, 2.18, 1.89, 1.67, and 0.88 mg malondialdehyde (MDA)-eq/kg for the control, BHT, *SO*EO, *SC*EO, and *SO*EO + *SC*EO samples, respectively, at day 10. These results confirm the similar effect of the EOs when used alone and their synergistic effect when mixed.

Monitoring lipid peroxidation by thiobarbituric acid reactive substance (TBARS) assay is a direct indicator of meat quality [77]. The main pathway of lipid peroxidation involves an autocatalytic chain reaction of free radicals. However, lipid peroxidation can also be catalyzed by various environmental factors, such as light or presence of oxygen, free radicals, and metal ions [76,78,79].

Previous investigations have shown a comparable effect in different meat systems. For example, *Salvia officinalis* EO, reduced TBARS levels in raw and cooked pork meatballs stored at 4 °C, and in fresh and frozen pork meatballs [80]. Sage EO 3% (*w*/*w*) significantly prevented lipid oxidation in raw pork and cooked beef [78]. Estévez et al. [81] found that 0.1% sage EO was more effective than 0.02% BHT in reducing MDA formation in liver pate for 90 days storage at 4 °C. Our findings, which are comparable to previous studies, suggest that Salvia EOs can be used as natural edible antioxidants to prevent oxidation in meat.

#### 2.4.2. Microbiological Evaluation

**Figure 3:**
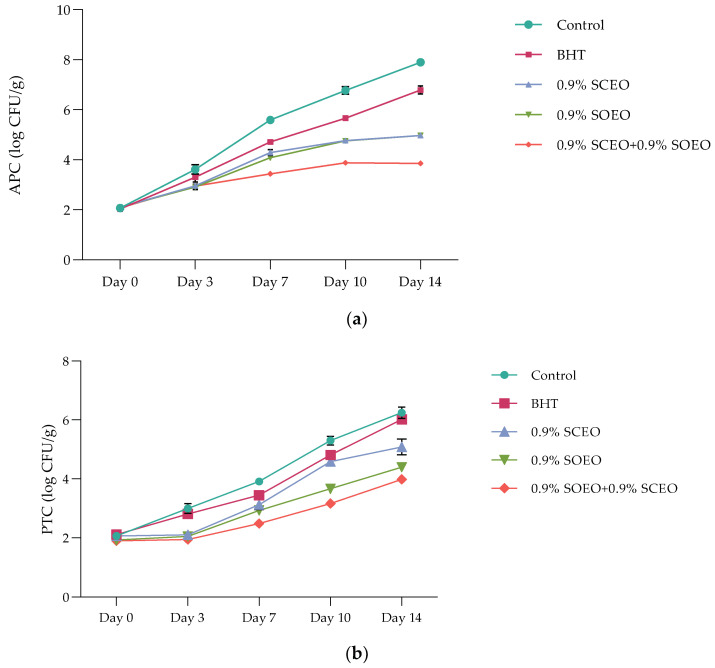
(**a**) Aerobic plate counts’ (APCs) evolution over time in treated and untreated minced beef meat samples; *Salvia officinalis* (*SO*EO) and *Salvia scarlea* (*SC*EO) essential oils and their combination (*SC*EO + *SO*EO) ± standard deviation (SD) of the three replicates; (**b**) Psychrotrophs counts’ (PTCs) evolution over time in treated and untreated minced beef meat samples; *Salvia officinalis* (*SO*EO) and *Salvia scarlea* (*SC*EO) essential oils and their combination (*SC*EO + *SO*EO) ± standard deviation (SD) of the three replicates.

Meat and meat products are ideal growth media for various microorganisms, some of which are pathogenic [82,83]. The results of microbiological analyzes are presented as described: aerobic plate counts (APCs) (Figure 3a), psychrotrophs counts (PTCs) (Figure 3b), and *Enterobacteriaceae* counts (ECs) (Table 5).

The APC profile of ground beef during 14 days of cold storage varied significantly from one treatment to another. The addition of *SO*EO and *SC*EO significantly improved the meat matrix compared to the synthetic antioxidant BHT and significantly decreased (*p* < 0.05) APC, with values of 4.94 ± 0.42, and 4.92 ± 0.31 log CFU/g for *SO*EO and *SC*EO, respectively. Probably, these data are due to the antimicrobial properties of sage, which can be attributed to the high content of monoterpene compounds, as reported above.

In mixtures of two EOs with equal proportions, APC reached a value of 3.91 ± 0.42 log CFU/g after 14 days, which extended the shelf-life of the minced meat by at least 10 days. However, it should be noted that both oils have antimicrobial effects and their synergistic action could increase the antimicrobial potential of food preservation.

Although the initial number of PTC was less than 2 log CFU/g after 7 days of incubation at 4 °C, the observed microbial growth is related to the temperature, high nutrient content, high water activity, and pH favorable for these microorganisms [84]. As expected, the initial PTC ranged from 5.38 ± 0.24 (control) to 3.02 ± 0.02 (*SO*EO + *SC*EO) log CFU/g, for all treatments, during the 14-days cold storage (Figure 3b). At the end of storage, the PTC differed significantly (*p* < 0.05) between the samples, which were divided as follows: control > BHT > *SO*EO ≥ *SC*EO > *SO*EO + *SC*EO. Therefore, the samples treated with *Salvia* EOs, showed satisfactory results for the preservation of fresh meat, and the PTC values did not reach the maximum limit for fresh meat, which was set at 6.0 log CFU/g [85].

The *Enterobacteriaceae* are a heterogeneous, Gram-negative group, some species of which are capable of fermenting lactose to produce acid and gas [84]. They are also known as coliform bacteria and are commonly used as indicator organisms in the food industry [86]. In microbiological quality testing, the number of *Enterobacteriaceae* is used as an effective parameter to evaluate the hygiene status and for possible failures during food processing [87]. The final step of the meat quality measurement was the monitoring of ECs during the five-time intervals of days 0, 3, 7, 10, and 14 (Table 5).

The beneficial effect of the addition of *SC*EO and *SO*EO (essentially as a mixture) was most evident in bacteria of the *Enterobacteriaceae* family, with differences of 1 to 2 log cycles between preserved and control samples. According to Hayouni et al. [88], the addition of *S. officinalis* EO to ground beef at a level of 0.02% was sufficient to produce a bacteriostatic effect that persisted over 15 days of storage at 4–7 °C. The reported results from present study also confirm that the addition of *SC*EO and *SO*EO can effectively control the growth of *Enterobacteriaceae*.

#### 2.4.3. Effective Action of Essential Oils on *Listeria monocytogenes* Inoculated into Minced Beef Meat

In this part of our work, we investigated the behavior of *L. monocytogenes* during inoculation of minced meat enriched with concentrations of 9.2% *SC*EO, *SO*EO and (*SC*EO + *SO*EO). First, as is well known, not all microbiologists agree that decontamination of meat is necessary or even desirable. Olaoye and Ntuen [89] considered that high concentrations of indigenous nonpathogenic microorganisms can have a protective effect on meat and meat products by displacing pathogens. However, our samples were decontaminated to reduce the number of factors involved in the growth of microorganisms in this nutritional model and to avoid interference with colonies on agar.

**Figure 4:**
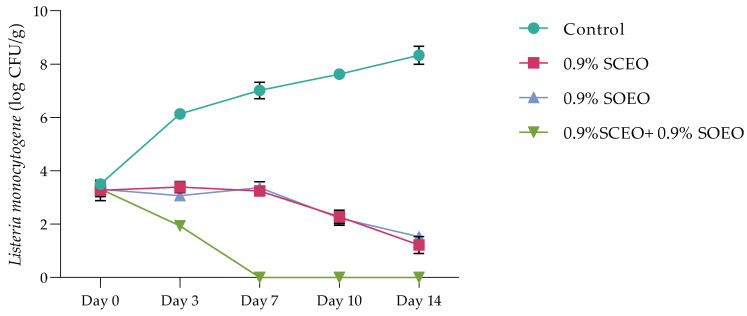
Time-dependent survival at 4 °C of Listeria monocytogene after treatment with *SC*EO (0.9%) and *SO*EO (0.9%) alone and in combination. Values are the mean of three individual replicates (means ± SD). Differences among the samples were determined using the Student’s *t*-test and were considered significant when *p* < 0.05, at a minimum.

The time-dependent survival rate of *L. monocytogenes* after treatment with *SC*EO and *SO*EO, is shown in Figure 4. The data from both treatments show a progressive decrease in the number of these bacteria, with both oils showing a similar effect with a non-significant difference (*p* > 0.05). The addition of a mixture of the two oils significantly reduced the number of *Listeria*, with a total bacteriostatic effect after 7 days, while the bacterial count in the control increased to 7.70 log CFU/mL after 14 days.

Similar results were obtained with *Salvia officinalis* EO (at a concentration of 0.2–0.5 µg/g) on beef experimentally contaminated with *B. cereus*, *S. aureus*, and *S. typhimurium* [89]. Previous studies have shown that both *SO*EO and *SC*EO are bacteriostatic against *S. anatum* and *S. enteritidis* at low concentrations [90].

#### 2.4.4. Sensory Evaluation of Beef Minced Meat

**Figure 5:**
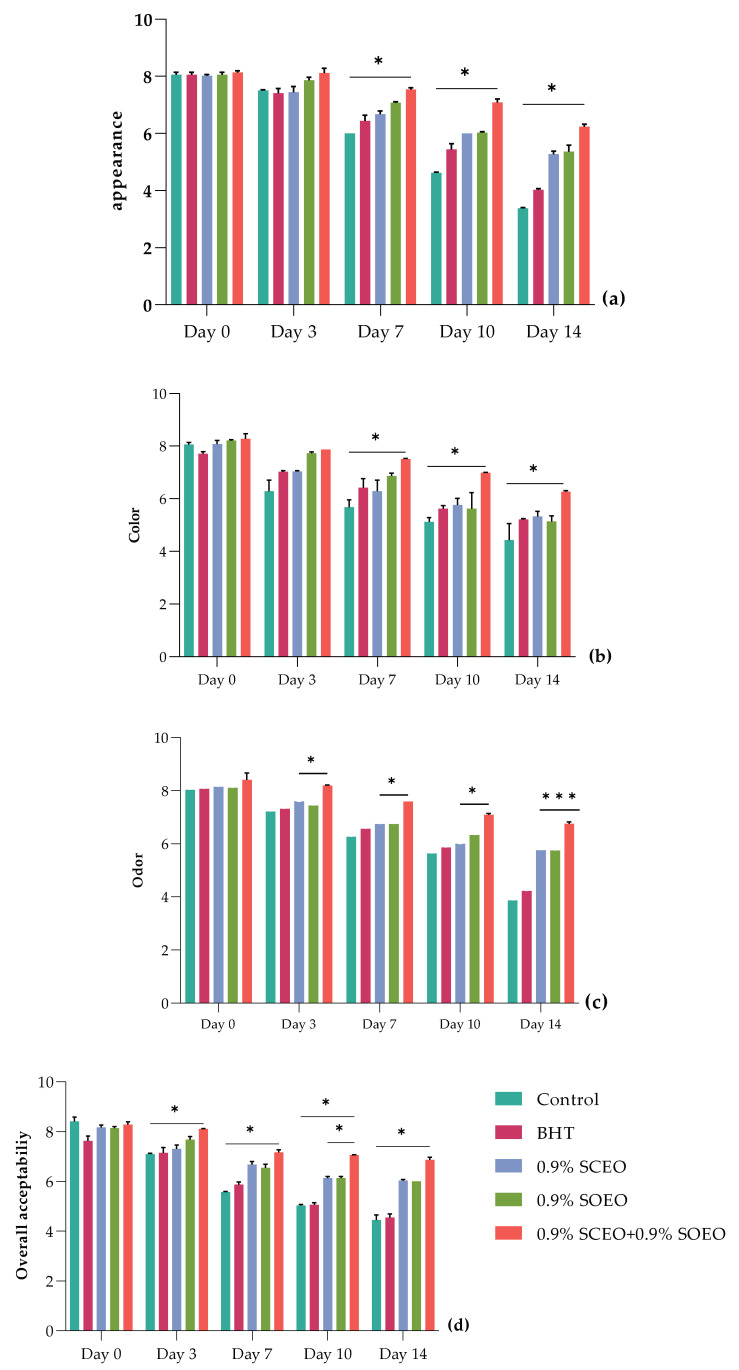
Sensor analysis for minced beef meat during 14 days of storage at 4 °C, including (**a**) appearance, (**b**) color, (**c**) odor, and (**d**) overall acceptability. Values are the mean of three individual replicates (means ± SD). Groups (BHT, 0.9% *SC*EO, 0.9% *SO*EO, 0.9% *SC*EO + 0.9% *SO*EO) vs. group (control): *** *p* ≤ 0.001, ** *p* ≤ 0.01, * *p* ≤ 0.05.

The effects of *SO*EO, *SC*EO and their mixture on sensory properties of chilled ground beef are shown in Figure 5. Sensory parameters, including appearance (Figure 5a), color (Figure 5b), odor (Figure 5c), and overall acceptability (Figure 5d), served as key indicators of potential consumer preferences. The results showed that longer storage (*p* < 0.05) significantly affected sensory quality in both control and treated samples.

As shown in Figure 5, the sensory characteristics of the minced meat were significantly (*p* < 0.05) affected by the different treatments during storage. The control and BHT samples showed a significant decrease (*p* < 0.05) in appearance, color, and overall acceptability parameters. In contrast to the untreated and BHT samples, treatment with *SC*EO and *SO*EO significantly improved odor, appearance, and general acceptability (*p* < 0.05), with the best values obtained for the mixture of the two oils because of their synergistic effect. In this regard, all three parameters were satisfactory in the samples treated with EOs until day 14 (*p* < 0.05), while they were undesirable in the untreated samples from day 10. In addition, the panelists evaluated the odor of the minced meat of samples treated with a rejection level of 5, which was not reached by the treatments with *SC*EO, *SO*EO, and *SC*EO + *SO*EO, with values of 5.70, 5.94, and 6.70, respectively [91].

For the other samples (BHT, *SC*EO, *SO*EO, and *SC*EO + *SO*EO), the color evaluation remained acceptable until the end of storage (Figure S3). It should be noted that these changes in sensory properties were related to oxidative changes associated with proteins and lipids and that these properties were improved by treatment with the EO mixture, as shown by the stability results after biochemical analysis [92,93].

Overall, the addition of *Salvia* EOs significantly improved the sensory characteristics of the minced meat during the investigated storage period. Similar trends were observed when sage was added to sausages at concentrations of 0.1% and 0.15%, reducing texture deterioration during cold storage (*p* < 0.05) [94]. Sage has been also shown to be effective in preserving the sensory properties of many foods, including fresh pork sausages, salmon burgers, and ground beef [88,93,94].

#### 2.4.5. Chemometric Approaches

**Figure 6:**
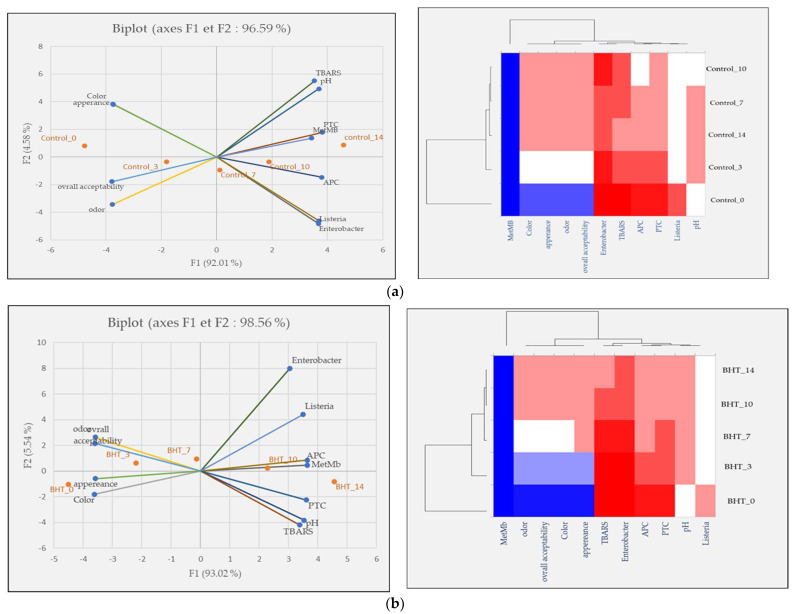

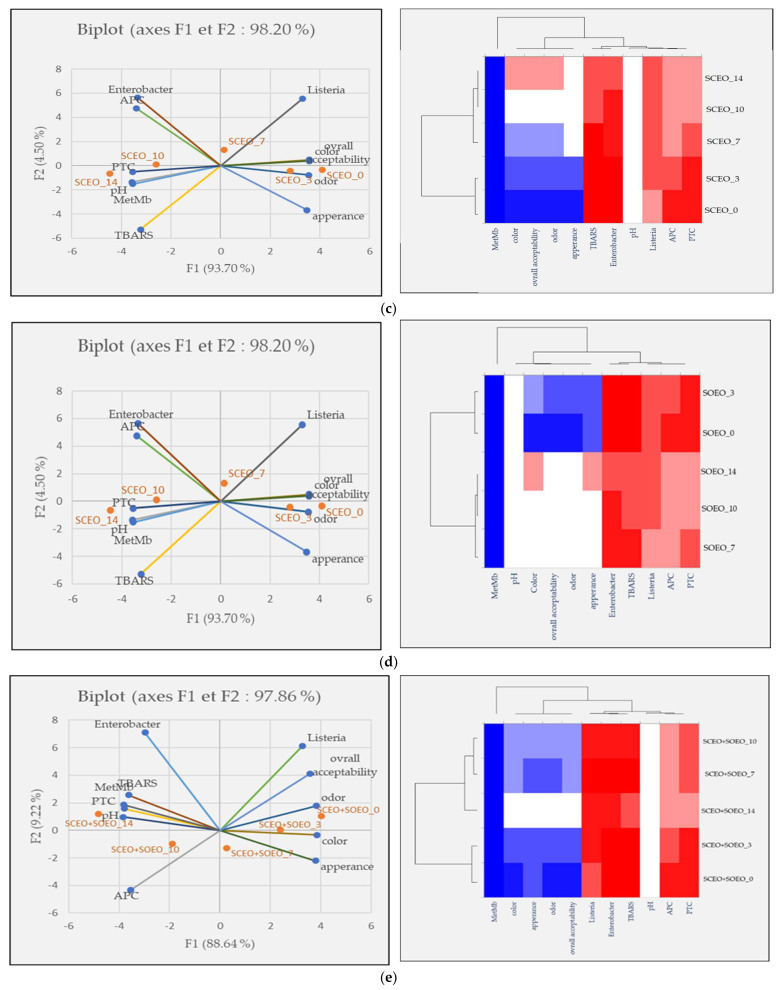
Classification of five samples throughout the storage period based on physicochemical properties, microbiological counts, and sensory parameters. Projection of variables by PCA, scatter plot for each meat sample over each storage period by PCA and a heat map of the same parameters for the (**a**) control sample; (**b**) BHT; (**c**) *SC*EO; (**d**) *SO*EO; (**e**) *SC*EO + *SO*EO.

Principal component analysis (PCA) was performed to investigate the relationship between the five samples over the five-time intervals, for meat quality measurements, and finally to show the effects of our treatments on ground meat quality. The score diagram shows the position of the objects in the multivariate space of the two principal component vectors. As can be seen, the variance obtained from the first two components (PCA1 and PCA2) was 96.59, 98.56, 98.20, 97.22, and 97.86% for the control, BHT, *SC*EO, *SO*EO, and *SC*EO + *SO*EO samples, respectively. In the view of similarities and differences, heat map diagrams for the evolution of each treatment over time were presented in Figure 6.

In Figure 6a, showing the effect of storage time on ground beef quality, a trend was observed to the right of PCA that correlated positively with higher values of TBARS, MetMb, pH, and PTC. The heat map confirms these results and proves the existence of a direct and positive correlation between the increase of MetMb and pH, bacterial contamination, and TBARS. This is in agreement with the findings of Mwove et al. [95], who investigated the physicochemical and sensory characteristics of beef roundels extended with gum arabic and found several significant correlations between beef roundels quality parameters. Similarly, Wang et al. [96] reported that lipid oxidation in meat products, is faster than oxidative protein degradation. From these results, it can be concluded that meat is a matrix that is very sensitive to oxidation processes and bacterial contaminations that affects its sensory attributes.

A similar trend in the change in scores was observed in the BHT-treated samples (Figure 6b): On days 0 and 3, the samples correlated positively with the best scores in the sensory analyses (color, odor, appearance, and overall acceptability). On the other hand, samples BHT_10 and BHT_14 showed very high levels of bacterial load and oxidation products.

Figure 6c,d show the results and heat maps of the samples treated with *SC*EO (9.2%) and *SO*EO (9.2%). The results of the two treatments are similar, with values moving from right to left, showing the conservative effect of our treatments.

For the mixture (Figure 6e) that showed the best results in terms of preservation efficiency, the distance between the observations on days 7, 10 and 14 was close or lower, proving the preservative effect of the mixture in slowing down the oxidation processes and bacterial contamination and, consequently, in preserving the sensory characteristics. In this context, Akacha et al. [37] showed that all sensory characteristics were correlated with the parameters of primary and secondary lipid oxidation (TBARS) and protein oxidation (MetMb%), as well as with microbial load.

All these characteristics are interdependent and important for the quality of the meat. Chemometric tools are widely used methods to evaluate the authenticity and quality of meat based on its oxidative stability and color characteristics during storage [97,98,99]. Our results show the effectiveness of *Salvia* EOs both alone and in effective mixture for meat preservation, ensuring microbiologically stable and safe meat without significantly affecting sensory quality. Therefore, the tested EOs could be used as natural preservatives in the food industry.

## 3. Materials and Methods

### 3.1. Materials

The essential oils from leaves of *Salvia officinalis* L. and *Salvia sclarea* growing in Tuscany, Italy and obtained by steam distillation, were directly provided by “èssenziale” Azienda Agricola, San Donato in Poggio (FI), Italy. The collection date of plant was in July 2022.

### 3.2. GC-MS Analyses of EOs

To describe the volatile chemical profile of the two Eos, a Clarus 500 model Perkin Elmer (Waltham, MA, USA) gas chromatograph coupled with a mass spectrometer and equipped with a flame detector ionization (FID), was used. The separation of compounds was performed by a Varian Factor Four VF-1 capillary column [100,101]. The oven temperature program started from 60 °C up to 220 °C for 20 min at a rate of 6 °C min^−1^. Helium was the carrier gas at flow rate of 1.0 mL min^−1^ in constant mode. For MS, the mass spectra were obtained in the electron impact mode (EI), at 70 eV in full-scan mode in the range 35–450 *m*/*z*. The compounds were identified by the matching their mass spectra with databases Wiley 2.2 (Wiley, NY, USA) and Nist 02 (Gaithersburg, MD, USA) and by comparing their linear retention indices (LRIs), relative to C_8_–C_25_ n-alkanes analyzed under the same conditions, with those available in the literature. The relative average percentages were calculated with respect to the total area of the chromatogram by normalizing the peak area without the use of an internal standard and any factor correction. All analyses were performed in triplicate.

### 3.3. Antioxidant Activity

#### 3.3.1. Phosphomolybdenum Assay

The antioxidant activity of EOs was evaluated according to the procedure described by Pervaiz et al. [102]. An aliquot of each sample (0.1 mL) was added to 1 mL of reagent solution (0.6 M sulfuric acid, 28 mM sodium phosphate, and 4 mM ammonium molybdate). The vial containing the mixture was sealed and incubated at 95 °C for 90 min. After incubation, the samples were cooled and their absorbance recorded at 765 nm. Percent inhibition was calculated using the following formula, while the IC_50_ was calculated using Graph Prism Pad software.

% inhibition = (1 − absorbance of sample/absorbance of control) × 100

#### 3.3.2. DPPH Radical Scavenging Activity

The antiradical activity of the EOs was evaluated according to Ben Hsouna et al. [103]. A 2 mL aliquot of the sample solution was mixed with 2 mL of 2,2-diphenyl-1-picrylhydrazyl (DPPH) solution (0.1 mM). The reaction mixtures were left at room temperature in the dark for 30 min after which their absorbances were recorded at 517 nm. Quercetin was used as a reference compound.

DPPH inhibition (%) = {(A0 − A1)/A0} ×100

where the A control and A sample were the measured absorbance of the control and sample, respectively.

### 3.4. Antibacterial Activity

#### 3.4.1. Microbial Strains

Authentic pure cultures of bacteria were obtained from the International Culture Collections: The American Type Culture Collection (ATCC) and the local culture collection of the Centre for Biotechnology of Sfax, Tunisia. They included Gram-positive bacteria: *Bacillus cereus* ATCC 14579, *Staphylococcus aureus* ATCC 25923, *Enterococcus faecalis* ATCC 29212, *Micrococcus luteus* ATCC 1880, and *Listeria monocytogenes* ATCC 19117, and Gram-negative bacteria: *Salmonella enterica* ATCC 43972, *Escherichia coli* ATCC 25922, and *Pseudomonas aeruginosa* ATCC 9027. Bacteria were cultured in Muller–Hinton agar (MHA) at 37 °C for 12–24 h, except for *Bacillus* species, which were incubated at 30 °C [104].

#### 3.4.2. Agar Diffusion Sensitivity Test

*SC*EO and *SO*EO were tested against all bacteria using the Kirby–Bauer disc diffusion test (sterile 9-mm paper discs; ANTF-009-1K0; PRAT DUMAS, Couze-St-Front, France). The inoculum (100 µL, 10^6^ CFU/mL) were spread over the entire surface of the MHA plate (Sifin Diagnostics GmbH, Berlin, Germany) using a Drigalski spatula. A sterile paper disc was placed in the center of a Petri dish [105]. Then, 80 μL of EO was added to the paper disc. The plates were incubated at 30 °C or 37 °C for 24 h. A digital caliper was used to measure the diameter of the zone of inhibition (in millimeters). Three replicates were performed for each EO.

### 3.5. Determination of Minimum Inhibitory and Bactericidal Concentrations of the EOs

The antibacterial assay was tested by the microdilution method [106] using 96-well microtiter plates to determine the minimum inhibitory concentration (MIC) and minimum bactericidal concentration (MBC). Bacterial suspensions were adjusted to a concentration of 1.0 × 10^6^ CFU/mL using sterile saline. The inoculum was prepared daily and stored at 4 °C until use. For validation of two EOs, they were dissolved in a 5% dimethyl sulfoxide solution (Merck KGaA, Darmstadt, Germany) containing 0.1% polysorbate-80 (1 mg/mL) and added to MHB (100 μL) containing bacterial inoculum (1.0 × 10^4^ CFU/well) to achieve the desired concentrations. The microtiter plates were incubated for 24 h at 37 °C on a rotary shaker (160 rpm). As an indicator of microbial growth, thiazolyl blue tetrazolium bromide (25 μL, 0.5 mg/mL) (Sigma-Aldrich, Taufkirchen, Germany) was used. MBC was defined as the lowest concentration required to kill 99% of bacteria [107] and it was calculated by taking 10 µL of the suspension from each well and inoculating it into Muller-Hinton string plates. The number of surviving organisms was determined after plates incubation at 37 °C for 24 h.

### 3.6. Analysis of Beef Meat Samples

**Figure 7:**
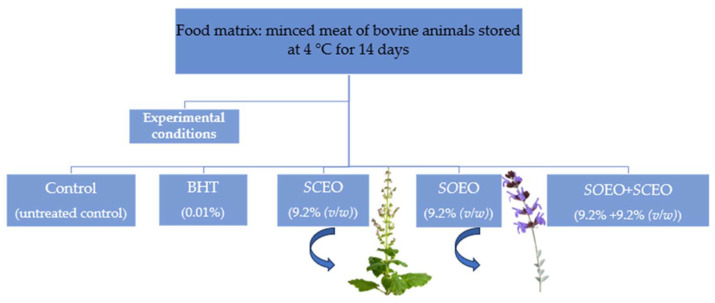
Experimental conditions for the preparation of samples stored at 4 °C with monitored parameters on days 0, 3, 7, 10, and 14.

Raw beef was purchased at a local market (Sfax, Tunisia). The samples were then minced with a meat grinder (10-mm plate followed by an 8-mm plate). They were placed on ice in insulated styrofoam boxes and brought to the laboratory within one hour of purchase. For packaging raw minced meat for storage at 4 °C, four equal portions (25 g each) were placed separately in sterile plastic bags. The samples were then divided into five batches, as shown in Figure 7.

#### 3.6.1. Physiochemical Analysis

##### pH Analysis

The pH was determined for the homogeneous mixtures of meat with distilled water in the ratio 1:10, *w*/*v* [18]. A minced meat sample (5 g) was homogenized in distilled water (50 mL, pH 7.00). The suspension was filtered, and the pH of the filtrate was measured using pH210 Microprocessor pH Meter (HANNA Instruments, Kehl am Rhein, Germany).

##### Evaluation of Protein and Lipid Oxidation

Metmyoglobin (MetMb) content was determined according to the method described by Dghais et al. [108]. Meat sample (5 g) was mixed with cold K_3_PO_4_ buffer (25 mL, 0.04 M, pH 6.8). The mixtures were homogenized and kept in an ice bath for 1 h, after which they were kept at 4 °C for 1 h. Subsequently, the samples were centrifuged at 4500 rpm for 30 min at 4 °C (Eltek MP-400-R, Eltek India, Delhi, India). The supernatant was collected and filtered through Whatman filter paper No. 42 (Whatman, Maidstone, UK). The absorbance was determined at 525 (A525), 572 (A572), and 700 (A700) nm. The percentage of MetMb was determined according to the formula of Wang et al. [109].

Thiobarbituric acid reactive substances (TBARS) values were determined following the work of Ben Akacha et al. [106]. The absorbance of the samples was quantified spectrophotometrically, and the results are expressed as mg of malonaldehyde (MDA) per kg of meat.

### 3.7. Microbiological Analysis

Ten grams of each sample was placed in a stomacher containing sterilized peptone water (90 mL) and homogenized. Decimal dilutions of the samples were then prepared and inoculated into the solid culture plate. Microbial counts were performed for (1) aerobic plate counts (APC) incubated at 30 °C for 48 h in plate counter agar (PCA) [110], (2) total psychrotrophic plate counts (PTC) incubated at 7 °C for 10 days in PCA [17], and (3) *Enterobacteriaceae* counts (EC) incubated at 37 °C for 24 h in violet red bile glucose agar [111].

#### Inhibitory Effect of *SC*EO, *SO*EO Alone and in Combination against Listeria Monocytogenes Inoculated into Minced Beef Meat

The in-situ efficacy of *SC*EO, *SO*EO and their combination against *L. monocytogenes* was evaluated in a ground beef model following the procedure described by Ben Hsouna et al. [15], with slight modifications. Briefly, a new working culture of *L. monocytogenes* at approximately 10^6^ CFU/mL was prepared by suspending 3 to 5 isolated colonies in 10 mL of Mueller-Hinton broth (MHB). Colonies were suspended in MHB and grown overnight at 37 °C for 24 h until stationary phase. Fresh lean bovine muscle from a slaughterhouse in Sfax-Tunisia was transported to the laboratory on ice in isolated Styrofoam boxes within one hour after cutting. To reduce the number of microorganisms on the surface of the beef muscle, each piece was immersed in boiling water for 5 min. The boiled surface of the muscle was removed with sterile knives under aseptic conditions. 25 ± 0.1 g of the thus prepared meat pieces were minced in a sterile meat grinder and placed in bags. Half of the meat samples were inoculated with 2 × 10^2^ CFU *L. monocytogenes*/g meat and mixed homogeneously for 3 min to ensure good distribution of the pathogen. Prior to inoculation of the second half of the meat samples, both *SO*EO and *SC*EO and their mixture were dissolved in 10% DMSO, filtered through black polycarbonate filters with a pore size of 0.22 μm (Millipore, Burlington, MA, USA), and then added to final concentrations of 9.2% of each EO in the meat and mixed to distribute the microorganisms uniformly.

All bags of meat samples were stored at 4 °C and analyzed for enumeration of *L. monocytogenes* after 0, 3, 7, 10, and 14 days by aseptically removing the pieces and mixing them with 250 mL of MHB. The samples were homogenized for 1 min and incubated at 37 °C for 6 h. After this pre-enrichment (to revive injured live cells), the remaining amount of *L. monocytogenes* was determined by plate colony counting.

After a 10-fold serial dilution with physiological saline, each sample (100 μL) was applied to the surface of a MHA medium and then incubated at 37 °C for 24 h. Sterile saline was added to the untreated control in place of the oil treatments, inoculated with the tested bacteria, and stored under the same conditions as the other samples. In all cases, three individual replicates of each experiment were performed.

### 3.8. Sensory Evaluation

Sensory evaluation of raw ground beef samples was tested by thirty laboratory panelists. Aroma, color, appearance, and overall acceptability were rated on a hedonic scale from 1 (very poor) to 9 (very good). All analyses were performed in triplicate and at regular intervals during 0, 3, 7, 10, and 14 days of cold storage [67].

### 3.9. Statistical Evaluation

All measurements were taken after 0, 3, 7, 10, and 14 days of storage, and trials were conducted with five treatments in a randomized complete block experiment. Three replicates were also performed for each storage period. Two-way analysis of variance (ANOVA) was performed for all variables, and in case of differences, means were compared using Tukey’s test at 5% significance level.

To group samples by microbial count, lipid/protein oxidation, and sensory parameters over the five storage periods, all variables were automatically scaled before applying chemometrics. Using XLSTAT for Windows (version 2022), PCA and heat maps were performed to distinguish the samples. Dendrograms were created to obtain a two-dimensional projection of the similarity or dissimilarity of the samples.

## 4. Conclusions

The results of this study show that *Salvia officinalis* and *Salvia sclarea* EOs, thanks to their particularly rich chemical composition in monoterpenes, have significant antimicrobial activity effective against all eight microorganisms tested, including *L. monocytogenes*. In addition to their antibacterial activity, both EOs have antioxidant properties comparable to those of synthetic antioxidants such as BHT and gallic acid. Therefore, thanks to their beneficial effects and as a source of bioactive metabolites, they can be used in many applications.

Improving the shelf-life of foods can have a significant economic impact by reducing losses due to spoilage. New trends in food preservation are leading to reduce the use of preservatives. In this context, both EOs have been used alone or in combination as natural preservatives in minced meat, with encouraging results as their use contributed to the reduction and elimination of experimentally inoculated *L. monocytogenes*. These preservative effects were further enhanced when we combined the two oils to extend the shelf-life of raw meat during cold storage. In-depth statistical analysis provided us with useful information to link oxidative and microbiological properties to sensory characteristics by using correlation models. In conclusion, the present study was a first attempt to demonstrate the efficacy of *SC*EO and *SO*EO as natural preservatives for meat and meat products.

## Figures and Tables

**Table 1:** Chemical volatile composition (percentage mean values ± SD) of *SO*EO and *SC*EO.

N°	Component ^1^	LRI ^2^	LRI ^3^	*SO*EO ^4^ (%)	*SC*EO ^5^ (%)
1	*α*-pinene	938	943	4.8 ± 0.03	Tr
2	camphene	941	946	6.9 ± 0.03	-
3	1-octen-3-ol	960	964	0.2 ± 0.01	-
4	*β*-pinene	973	980	14.5 ± 0.08	-
5	*β*-myrcene	982	987	-	0.5 ± 0.02
6	*α*-terpinene	1018	1020	0.1 ± 0.00	-
7	*p*-cymene	1028	1026	0.2 ± 0.01	-
8	*trans*-*β*-ocimene	1051	1048	0.2 ± 0.02	0.1 ± 0.00
9	limonene	1031	1029	2.4 ± 0.02	0.1 ± 0.01
10	1,8-cineole	1035	1033	2.6 ± 0.02	-
11	*γ*-terpinene	1051	1048	0.4 ± 0.01	-
12	*trans*-sabinene hydrate	1055	1053	0.2 ± 0.02	-
13	*α*-ocimene	1060	1057	-	0.4 ± 0.02
14	terpinolene	1090	1087	0.3 ± 0.01	-
15	linalool	1095	1092	0.3 ± 0.01	11.3 ± 0.05
16	*α*-thujone	1105	1097	12.9 ± 0.06	-
17	camphor	1141	1139	19.0 ± 0.07	0.1 ± 0.01
18	*trans*-3-pinanone	1145	1141	0.8 ± 0.01	-
19	L-borneol	1150	1152	3.4 ± 0.03	-
20	terpinen-4-ol	1178	1182	0.4 ± 0.02	2.0 ± 0.02
21	linalyl formate	1201	1206	-	0.1 ± 0.01
22	*cis*-geraniol	1230	1227	-	0.4 ± 0.02
23	linalyl acetate	1255	1252	-	59.3 ± 1.14
24	bornyl acetate	1287	1290	5.6 ± 0.02	0.2 ± 0.01
25	nerol acetate	1322	1326	-	1.5 ± 0.02
26	*α*-cubebene	1352	1350	0.3 ± 0.01	-
27	geranyl acetate	1371	1366	-	2.5 ± 0.02
28	*α*-copaene	1381	1379	0.8 ± 0.02	1.2 ± 0.02
29	(-)-*β*-bourbonene	1390	1388	0.1 ± 0.00	-
30	*β*-caryophyllene	1445	1440	1.2 ± 0.04	3.7 ± 0.02
31	*γ*-gurjunene	1447	1444	-	0.8 ± 0.02
32	aromadendrene	1463	1460	3.3 ± 0.06	0.4 ± 0.01
33	humulene	1477	1473	11.9 ± 0.08	1.7 ± 0.03
34	*γ*-muurolene	1490	1486	1.6 ± 0.03	-
35	germacrene D	1492	1489	0.3 ± 0.02	10.5 ± 0.04
36	ledene	1498	1496	1.6 ± 0.03	-
37	bicyclogermacrene	1506	1504 *	-	0.8 ± 0.03
38	*δ*-cadinene	1511	1509 *	1.6 ± 0.02	0.7 ± 0.02
39	*α*-calacorene	1531	1528	Tr	-
40	spathulenol	1567	1563	0.7 ± 0.03	0.3 ± 0.02
41	globulol	1581	1576	0.1 ± 0.02	-
42	caryophyllene oxide	1583	1580	0.2 ± 0.02	0.2 ± 0.02
43	humulene epoxide II	1615	1611	0.6 ± 0.02	0.3 ± 0.02
44	*α*-eudesmol	1655	1650	-	0.1 ± 0.01
45	*β*-eudesmol	1657	1652	-	0.1 ± 0.00
46	*α*-cadinol	1682	1676	Tr	-
47	geranyl-*p*-cymene	1996	1993	-	0.6 ± 0.03
	SUM			99.5	99.9
	Monoterpene hydrocarbons			54.9	71.9
	Oxygenated monoterpenes			20.3	6.4
	Sesquiterpene hydrocarbons			21.1	19.8
	Oxygenated sesquiterpene			1.6	1.0
	Others			0.6	0.8

^1^ The components are reported according to their elution order on the apolar column. ^2^ Linear retention indices calculated using the apolar column. ^3^ Linear retention indices from the literature. * Normal alkane retention index. ^4^ Percentage mean values of *Salvia officinalis* EO components. ^5^ Percentage mean values of vehiculated *Salvia sclarea* EO components. Tr: percentage mean values < 0.1%; -: not detected.

**Table 2:** Antibacterial activity by diffusion test of *Salvia officinalis* (*SO*EO) and *Salvia scarlea* (*SC*EO).

	Diameter of Inhibition Zones (mm)			
	*SO*EO		*SC*EO	
Concentration (µg/mL)	25.0	50.0	25.0	50.0
Gram positive				
*Bacillus cereus* ATCC 14579	-	-	17.0 ± 0.71	23.0 ± 0.02
*Staphylococcus aureus* ATCC 25923	12.0 ± 0.04	14.0 ± 0.12	17.0 ± 0.01	21.0 ± 0.45
*Enterococcus faecalis* ATCC 29212	8.0 ± 0.14	9.0 ± 0.23	14.0 ± 0.24	17.0 ± 0.22
*Micrococcus luteus* ATCC 1880	12.0 ± 0.12	14.0 ± 0.75	19.0 ± 0.14	25.0 ± 0.21
*Listeria monocytogenes* ATCC 1911	-	-	17.0 ± 0.12	20.0 ± 0.42
Gram negative				
*Pseudomonas aeruginosa* ATCC 9027	-	-	-	-
*Escherichia coli ATCC* 25922	-	-	-	-
*Salmonella enterica* ATCC 43972	-	15.0 ± 0.01	-	-

±: Standard deviation (SD) of three replicates.

**Table 3:** The antibacterial activity of *Salvia officinalis* and *Salvia scarlea* EOs was evaluated against eight food-related pathogenic strains detected as minimum inhibitory concentration (MIC) and minimum bactericidal concentration (MBC) (mg/mL).

Bacterial Strains	MIC	MBC	MBC/MIC	Interpretation
*Salvia officinalis*				
Gram positive				
*Bacillus cereus* ATCC 14579 *Staphylococcus aureus* ATCC 25923	5.6 ± 0.68 5.6 ± 0.68	11.2 ± 0.3 11.2 ± 0.3	2 2	Bactericidal Bactericidal
*Enterococcus faecalis* ATCC 29212 *Micrococcus luteus* ATCC 1880	5.6 ± 0.68 4.6 ± 0.03	22.5 ± 0.6 11.5 ± 0.3	4 2	Bacteriostatic Bactericidal
*Listeria monocytogenes* ATCC 1911	4.6 ± 0.03	15 ± 0.0	3	Bacteriostatic
Gram negative				
*Pseudomonas aeruginosa* ATCC 9027	7.5 ± 0.00	11.2 ± 0.3	0.5	Bactericidal
*Escherichia coli ATCC* 25922	7.5 ± 0.31	11.2 ± 0.3	1	Bactericidal
*Salmonella enterica* ATCC 43972	4.6 ± 0.03	11.2 ± 0.3	0.7	Bactericidal
*Salvia sclarea*				
Gram positive				
*Bacillus cereus* ATCC 14579 *Staphylococcus aureus* ATCC 25923	7.5 ± 0.00 5.6 ± 0.68	15 ± 0.0 15 ± 0.0	2 2	Bactericidal Bactericidal
*Enterococcus faecalis* ATCC 29212 *Micrococcus luteus* ATCC 1880	7.5 ± 0.00 7.5 ± 0.00	18.7 ± 0.9 15 ± 0.0	2 2	Bactericidal Bactericidal
*Listeria monocytogenes* ATCC 1911	4.6 ± 0.31	18.7 ± 0.9	4	Bacteriostatic
Gram negative				
*Pseudomonas aeruginosa* ATCC 9027	11.2 ± 0.31	15 ± 0.0	1	Bactericidal
*Escherichia coli* ATCC 25922	3.7 ± 0.00	22.5 ± 0.6	6	Bacteriostatic
*Salmonella enterica* ATCC 43972	7.5 ± 0.00	22.5 ± 0.6	3	Bacteriostatic

±: Standard deviation (SD) of three replicates.

**Table 4:** Effect of *Salvia officinalis* (*SO*EO) and *Salvia sclarea* (*SC*EO) essential oils and their combination on the pH evolution of minced beef stored at 4 °C for 14 days.

Samples	pH Trend During the Days of Refrigerated Storage at 4 °C				
	0	3	7	10	14
**Control**	5.15 ± 0.07 ^aA^	5.58 ± 0.00 ^bC^	5.89 ± 0.04 ^cC^	6.62 ± 0.05 ^Dd^	7.85 ± 0.05 ^eD^
**BHT**	5.25 ± 0.06 ^aA^	5.32 ± 0.14 ^bB^	5.72 ± 0.08 ^bcBC^	6.29 ± 0.05 ^cC^	6.96 ± 0.08 ^cC^
***SC*EO**	5.10 ± 0.13 ^aA^	5.32 ± 0.14 ^bB^	5.65 ± 0.03 ^bB^	6.09 ± 0.01 ^cB^	6.52 ± 0.02 ^cB^
***SO*EO**	5.30 ± 0.014 ^aA^	5.41 ± 0.28 ^aB^	5.63 ± 0.01 ^bB^	6.01 ± 0.02 ^bB^	6.46 ± 0.02 ^bB^
***SC*EO + *SO*EO**	5.19 ± 0.07 ^aA^	5.25 ± 0.07 ^aA^	5.43 ± 0.08 ^aA^	5.67 ± 0.02 ^aA^	5.94 ± 0.05 ^bA^

±: Standard deviation (SD) of three replicates; a–d: mean values within all the samples not followed by a similar letter in the same column varied significantly (*p* < 0.05); A–D: mean values during storage not followed by a similar letter in the same line varied significantly (*p* < 0.05).

**Table 5:** Enumeration of *Enterobacteriaceae* in various samples of raw minced beef meat stored at 4 °C for 14 days.

Samples	Days of Refrigerated Storage				
	0	3	7	10	14
*Enterobacteriaceae* Counts (log CFU/g)					
**Control**	<1 ^aA^	1.25 ± 0.02 ^bAB^	2.25 ± 0.06 ^bB^	2.79 ± 0.24 ^dBC^	3.36 ± 0.07 ^aC^
**BHT**	<1 ^aA^	1.06 ± 0.06 ^bAB^	1.99 ± 0.01 ^abB^	2.52 ± 0.18 ^cBC^	2.84 ± 0.15 ^aC^
***SC*** **EO**	<1 ^aA^	<1 ^aA^	1.33 ± 0.91 ^bAB^	1.70 ± 0.10 ^cB^	2.11 ± 0.02 ^cC^
***SO*** **EO**	<1 ^aA^	1.24 ± 0.19 ^aAB^	1.92 ± 0.14 ^abAB^	2.01 ± 0.03 ^bBC^	2.26 ± 0.1 ^bC^
***SC*** **EO + *SO*EO**	<1 ^aA^	<1 ^aA^	<1 ^aA^	<1 ^aA^	1.68 ± 0.29 ^aB^

*Salvia officinalis* (*SO*EO, 0.9%) and *Salvia scarlea* (*SC*EO, 0.9%) essential oils and their combination (*SC*EO + *SO*EO, 0.9% + 0.9%); ±: standard deviation (SD) of three replicates; a–d: mean values within all the samples not followed by a similar letter in the same column varied significantly (*p* < 0.05); A–C: mean values during storage not followed by a similar letter in the same line varied significantly (*p* < 0.05).

## Data Availability

All generated data are included in this article.

## Supplementary Materials

The following supporting information can be downloaded at: https://www.mdpi.com/article/10.3390/plants12193385/s1, Figure S1: GC-FID Chromatogram of *SO*EO. Figure S2: GC-FID Chromatogram of *SC*EO. Figure S3: An example of visual aspects, after 7 days of storage at 4 °C, of ground beef treated with different concentrations of *SO*EO.
