# Supplementary material for: Salvia officinalis L. and Salvia sclarea Essential Oils: Chemical Composition, Biological Activities and Preservative Effects against Listeria monocytogenes Inoculated into Minced Beef Meat
Source: Plants (Basel). 2023 Sep 25;12(19):3385. doi: 10.3390/plants12193385 (PMC10574192; doi:10.3390/plants12193385)
Supplement: Supplementary file 1 [file plants-12-03385-s001.zip › plants-2609979-supplementary.pdf]

plants

Supplementary Material

Salvia officinalis L. and *Salvia sclarea* essential oils: chemical composition, biological activities and preservative effects against *Listeria monocytogenes* inoculated into minced beef meat

Boutheina Ben Akacha[1], Anis Ben Hsouna[1,2], Ivana Generalić Mekinić[3], Améni Ben Belgacem[1], Rania Ben Saad[1], Wissem Mnif[4], Miroslava Kačániová[5], Stefania Garzoli*[6].

1 Laboratory of Biotechnology and Plant Improvement, Centre of Biotechnology of Sfax, B.P "1177", Sfax 3018, Tunisia; akachabouthaina@gmail.com (B.B.A.), amanibelgassem@gmail.com (A.B.B.)
2 Department of Environmental Sciences and Nutrition, Higher Institute of Applied Sciences and Technology of Mahdia, University of Monastir, Monastir 5000, Tunisia; benhsounanis@gmail.com (A.B.H).
3 Department of Food Technology and Biotechnology, Faculty of Chemistry and Technology, University of Split, R. Boškovića 35, HR-21000 Split, Croatia; gene@ktf-split.hr (I.G.M).
4 Department of Chemistry, College of Sciences at Bisha, University of Bisha, P.O. Box 199, Bisha 61922, Saudi Arabia; wmoneef@ub.edu.sa (W.M.).
5 Institute of Horticulture, Faculty of Horticulture, Slovak University of Agriculture, Tr. A. Hlinku 2,949 76 Nitra, Slovakia; miroslavakacaniova@gmail.com (M.K.).
6 Department of Chemistry and Technologies of Drug, Sapienza University, P. le Aldo Moro, 5,00185 Rome, Italy; stefania.garzoli@uniroma1.it (S.G.)

* Correspondence: stefania.garzoli@uniroma1.it;

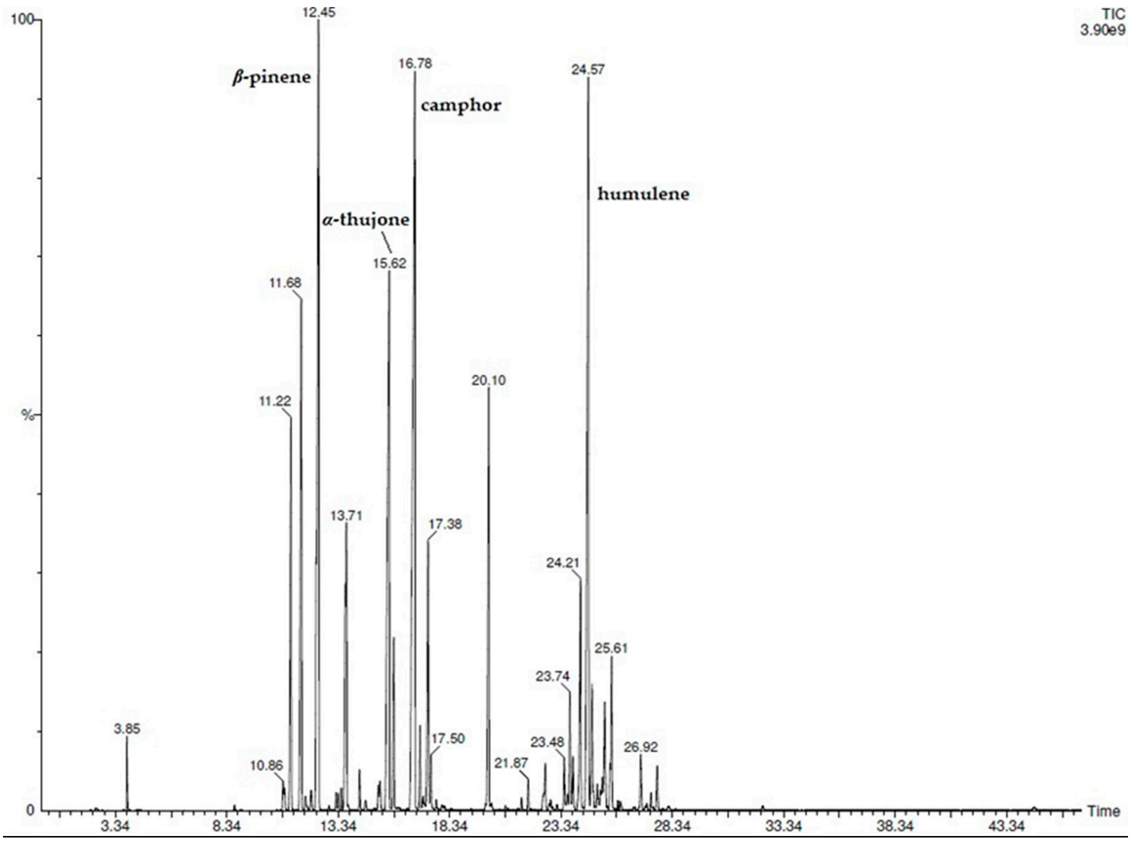

Figure S1. GC-FID Chromatogram of *SO*EO

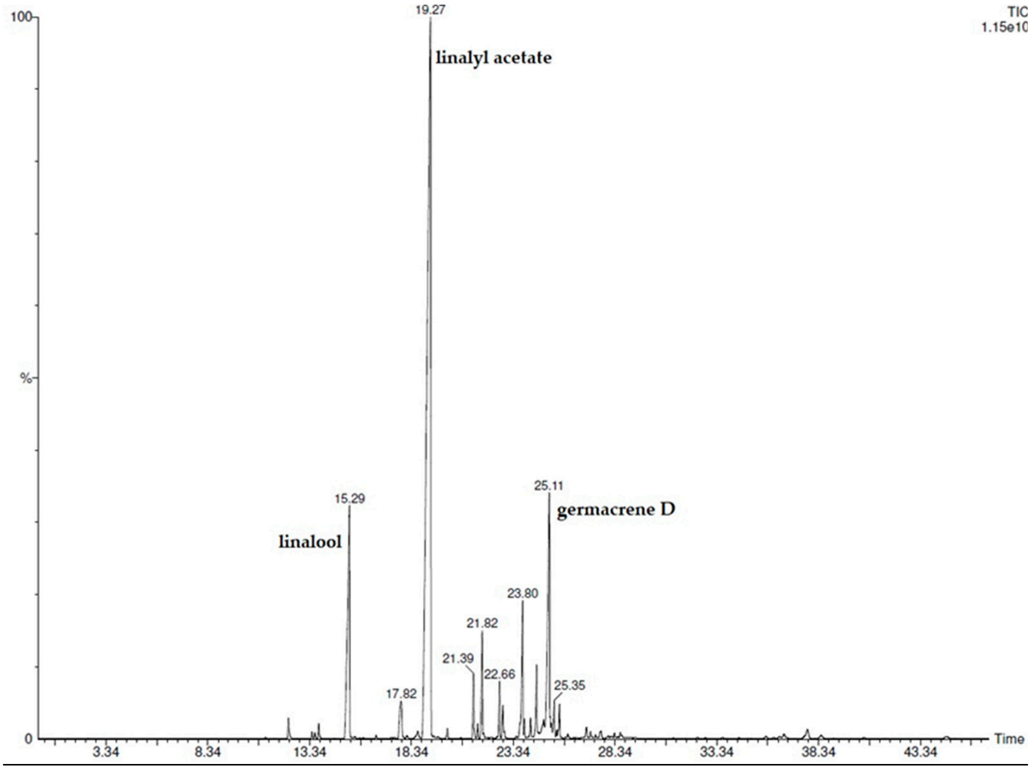

Figure S2. GC-FID Chromatogram of *SC*EO

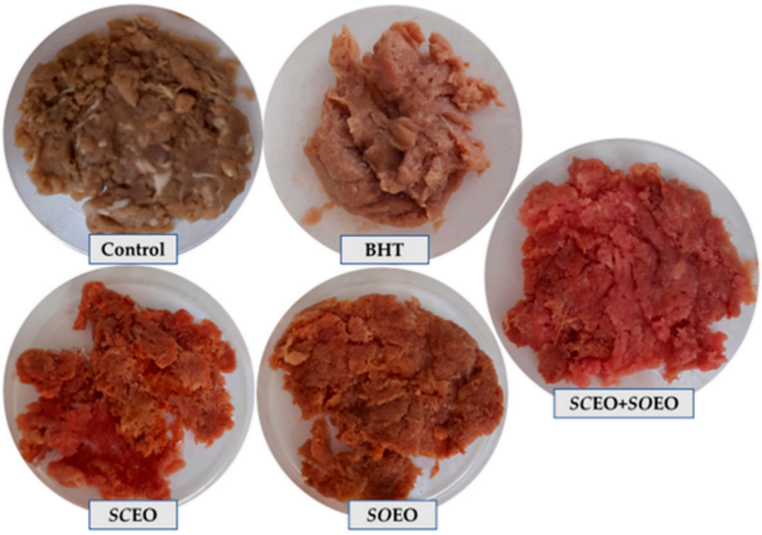

Figure S3. An example of visual aspects, after 7 days of storage at 4°C, of ground beef treated with different concentrations of *SO*EO
